# Impact of Autolysed Brewer’s Yeast and Soluble Dried Yeast Extract on Growth Performance and Mucosal Health of Atlantic Salmon (*Salmo salar*) Parr

**DOI:** 10.3390/ani15030323

**Published:** 2025-01-23

**Authors:** Sheu G. Odu-Onikosi, Taofik A. Momoh, Sherilyn T. Abarra, Noah E. Wood, Folasade D. Amulejoye, Matthew Emery, Glenn M. Harper, Benjamin Eynon, Victor Kuri, Holger Kühlwein, Daniel L. Merrifield

**Affiliations:** 1 Fish Health and Nutrition Research Group, School of Biological and Marine Sciences, University of Plymouth, Plymouth PL4 8AA, UK; taofik.momoh@plymouth.ac.uk (T.A.M.); sherilyn.abarra@plymouth.ac.uk (S.T.A.); noahedwardwood@outlook.com (N.E.W.); matthew.emery@plymouth.ac.uk (M.E.); glenn.harper@plymouth.ac.uk (G.M.H.); ben.eynon@plymouth.ac.uk (B.E.); v.kuri@plymouth.ac.uk (V.K.); 2 Department of Fisheries and Aquaculture, Lagos State University of Science and Technology, Ikorodu 104101, Nigeria; 3 Department of Fisheries and Aquaculture Technology, Olusegun Agagu University of Science and Technology, P.M.B. 353, Okitipupa 350105, Nigeria; fd.amulejoye@oaustech.edu.ng; 4 Leiber GmbH, 49565 Bramsche, Germany; h.kuehlwein@leibergmbh.de

**Keywords:** Atlantic salmon parr, autolysed brewer’s yeast, soluble dried yeast extract, gut microbiota, immune response

## Abstract

Yeast-based feed additives have emerged as promising functional feed additives (FFAs) to promote sustainable aquaculture development through enhanced gut health and immune modulation in fish. The present study evaluated the impact of autolysed brewer’s yeast (ABY) and soluble dried yeast extract (SDYE) in improving the intestinal and skin histology, immune response, and intestinal microbiome of Atlantic salmon parr (*Salmo salar*) over a 9-week feeding trial. Three experimental diets were produced: a control diet, a diet supplemented with ABY at 2.5 g/kg, and a diet supplemented with SDYE at 2.5 g/kg. These diets were administered to triplicate tanks of Atlantic salmon. The yeast-supplemented diets, especially ABY, improved intestinal mucosal fold length, lamina propria width, microvilli density, and intestinal goblet cell counts, as well as skin goblet cell counts. The yeast additives had no detrimental effects on the fish haematology, with no significant differences in haemoglobin concentration, red blood cell counts, and white blood cell counts among the treatment groups. Gene expression analysis revealed upregulation of *il-1β* and *muc-2* in fish fed the ABY diet, indicating enhanced immune function and potentially mucosal protection. Intestinal microbiota analysis revealed *Firmicutes* as the most dominant phylum in all groups, followed by *Actinobacteriota.* Distinct bacterial community shifts were observed between the treatment groups, with a significant increase in the relative abundance of taxa such as *Staphylococcus* in yeast-supplemented diets and a significant decrease in *Streptococcus* and *Weissella*. Collectively, these findings suggest that the yeast additives, especially ABY, enhance gut health and immune function without compromising growth performance.

## Simple Summary

The aquaculture industry is increasingly pursuing sustainable practices, including the use of functional feed additives (FFAs) to enhance fish health and growth, and reduce reliance on antibiotics usage in aquafeeds. Brewer’s yeast-derived additives have emerged as promising FFAs, showing significant potential in promoting sustainable aquaculture development. In this study, the use of commercial brewer’s yeast-derived feed additives was explored to improve the health and performance of Atlantic salmon. The salmon were fed diets supplemented with autolysed brewer’s yeast (ABY) or soluble dried yeast extract (SDYE) for nine weeks to assess their impact on skin and intestinal health, immune response, intestinal bacterial communities, and growth. The results showed that the brewer’s yeast additives, particularly ABY, improved intestinal structure, enhanced immune-related gene expression, and positively influenced the bacterial community in the intestine, all while maintaining healthy blood parameters and growth rates. These findings suggest that the yeast additives can support fish health and resilience, offering a sustainable way to boost aquaculture productivity while reducing reliance on antibiotics. This has potential benefits for both environmental sustainability and global food security.

## 1. Introduction

Atlantic salmon (*Salmo salar*) is an important species in global aquaculture. With Norway, Chile, the United Kingdom, and Canada leading production, Atlantic salmon ranks as one of the most valuable farmed finfish, reaching 2.87 million metric tonnes in global output, and valued at USD 21.92 billion in 2022 [1]. In addition to its economic significance, Atlantic salmon is a valuable source of omega-3 fatty acids, which are essential nutrients for human health [2].

As the aquaculture industry pursues more sustainable practices, there is growing interest in identifying functional feed additives (FFAs) that can be optimised for different fish species at various stages of development. Studies have shown that FFAs can enhance fish disease resilience, improve nutrient absorption, and reduce dependence on antibiotics, contributing to more sustainable production systems [3,4]. Brewer’s yeast and its derivative products are particularly promising FFAs due to their rich content of bioactive compounds like β-glucans, mannan oligosaccharides (MOS), nucleotides, and peptides, which are known to support fish growth, modulate immune responses, enhance gut health, and influence gut microbial diversity [5,6,7].

Mucosal health plays a vital role in maintaining the overall well-being of fish by serving as the first line of defence against pathogens and environmental stressors [8,9]. Enhancing mucosal health through diet, particularly with the inclusion of functional feed additives, can improve epithelial barrier integrity, boost goblet cell production, and modulate immune-related gene expression, ultimately leading to better disease resistance and growth performance [10,11]. As such, interventions targeting mucosal health not only contribute to individual fish resilience but also enhance the productivity and sustainability of aquaculture systems.

Several studies have reported the beneficial effects of yeast-derived additives, especially β-glucans and MOS, on Atlantic salmon. These additives have been associated with improved growth performance [12], favourable changes in gut morphology [13,14], and enhanced mucosal immunity [15], as well as modulated gut microbial communities [16]. Despite these promising findings, research on the specific effects of other brewer’s yeast-derived products, such as fully autolysed brewer’s yeast (ABY) and soluble dried yeast extract (SDYE), on gut health in Atlantic salmon parr remains limited.

This study investigates the effects of two commercial yeast additives, an autolysed brewer’s yeast (CeFi^®^ Pro, Leiber GmbH, Bramsche, Germany) and a soluble dried yeast extract (SDYE, Leiber GmbH, Bramsche, Germany), on the growth performance and mucosal health of Atlantic salmon parr. ABY is produced through gentle autolysis, retaining both the yeast cell wall and the cell interior, and contains bioactive substances such as β-glucans, MOS, nucleotides, and peptides. In contrast, SDYE is produced by detaching the yeast cell interior from the yeast cell wall, and comprises only the soluble components of the cell interior, such as nucleotides and peptides. Due to their bioactive components, these two yeast additives are hypothesised to improve growth and mucosal health in Atlantic salmon parr. By assessing histological, haematological, and immunological impacts and analysing microbial community shifts associated with these dietary supplements, this study primarily aims to determine their efficacy in enhancing the overall health of Atlantic salmon parr under experimental conditions.

## 2. Materials and Methods

### 2.1. Experimental Design and Fish

The feeding trial was carried out at the University of Plymouth in a cold freshwater recirculating aquaculture system (RAS) of the Fish Health and Nutrition Research Group. The RAS system consisted of 9 rectangular fibreglass tanks. The water volume in each tank was maintained at 70 L and recirculated at a rate of 900 L per hour. Each tank was supplied with continuous aeration, and the water quality parameters were closely monitored and maintained to ensure they remained within a suitable range for Atlantic salmon parr. The Atlantic salmon parr were purchased from Landcatch Ltd. in Scotland, United Kingdom, and were acclimated in the experimental system for 2 weeks before starting the experiment. After acclimation, 180 Atlantic salmon parr (37.08 ± 0.10 g) were randomly allocated into 9 experimental tanks, with 20 fish allotted to each tank. The average water temperature was 15.64 ± 0.27 °C, pH 6.76 ± 0.25, dissolved oxygen 9.99 ± 0.22 mg/L, nitrate 24.86 ± 6.32 mg/L, nitrite 0.02 ± 0.01 mg/L, and ammonium 0.02 ± 0.01 mg/L. Throughout the trial and during acclimation, the Atlantic salmon parr were subjected to a fixed light and dark photoperiod of 12 h each.

### 2.2. Experimental Diets and Feeding

The yeast additives investigated included an autolysed brewer’s yeast (ABY; CeFi^®^ Pro) and a soluble dried yeast extract (SDYE) produced and supplied by Leiber GmbH, Bramsche, Germany. The CeFi^®^ Pro is a specially dried autolysed brewer’s yeast feed ingredient (100% *Saccharomyces cerevisiae*) consisting of both cell wall components (predominantly ß-glucans and mannans) and soluble cell content (nucleotides, peptides, amino acids, etc.). The cell wall of the ABY (CeFi^®^ Pro) is partially perforated, and the cell content is broken down into smaller fragments due to the autolysis process, thereby increasing the bioavailability and bioactivity. The SDYE is a soluble dried brewer’s yeast extract feed ingredient (100% *Saccharomyces cerevisiae*) consisting of soluble cell content only. The nutritional profile of the two yeast additives is shown in Table 1.

Three isonitrogenous and isolipidic diets, containing approximately 48% protein and 24% lipid, were formulated to meet the established nutrient requirements for Atlantic salmon parr [17] using Animal Feed Optimisation Software (AFOS) Cloud. The experimental diets were produced in the Nutrition and Product Development Laboratory at the University of Plymouth using commercially relevant raw materials (Table 2). The experimental diets included a basal diet (control) and two diets supplemented with brewer’s yeast additives, the ABY diet (2.5 g/kg) and the SDYE diet (2.5 g/kg). The proximate composition of the diets (Table 2) was determined following AOAC protocols [18]. The nitrogen-free extract was calculated by subtracting the sum of crude protein, lipid, moisture, and ash from 100, while the gross energy was estimated by applying the conversion factors for protein (5.65 kcal/g), lipid (9.45 kcal/g), and NFE (4.10 kcal/g) [19].

Each experimental diet was tested in triplicate over a 9-week feeding trial, with fish hand-fed their respective diet daily at 1% of body weight. The daily ration was divided into three equal portions and administered at 9:30, 13:00, and 16:30 h. Weekly, the total fish biomass in each tank was assessed by weighing all fish collectively, allowing the feed input to be recalculated and adjusted based on updated total body weight.

### 2.3. Fish Sampling

A total of 6 representative fish from each tank (18 per treatment) were collected, euthanised with an overdose of buffered MS222 (Sigma-Aldrich, Poole, UK) solution (200 mg/L water for 5 min), and brain pithed following the UK Home Office Schedule 1 guidelines. The MS222 solution was buffered with sodium bicarbonate to maintain the neutral pH of the water and reduce stress during euthanasia. For 3 of these fish per replicate (9 per treatment), length and weight were recorded to calculate the Fulton’s condition factor. Blood, skin (from the dorsal region, just above the lateral line), and posterior intestine (PI) samples were collected from this set for analyses.

Blood samples (ca 50 µL) were drawn from the caudal vein with a 25-gauge needle and 1 mL syringe, and transferred into a 1.5 mL microcentrifuge tube. From this, 4 µL was pipetted into a tube with 1 mL of Drabkin’s solution to determine haemoglobin concentration, while 20 µL was added to a tube with 980 µL of Dacie’s solution for red and white blood cell counts. Skin and PI samples were excised for histological analysis. Skin and PI samples (ca 5 mm) were prepared for light microscopy, while only PI samples (ca 2 mm) were analysed with electron microscopy. Tissues for light microscopy were fixed in 10% natural buffered formalin and stored at 4 °C for 48 h before transferring them to 70% ethanol for extended storage at room temperature. Samples for electron microscopy (scanning and transmission) histological analysis were fixed in 2.5% glutaraldehyde, with those for scanning electron microscopy (SEM) rinsed in 1% S-carboxymethyl-L-cysteine prior to fixation. For gene expression analysis, PI samples (ca 200 mg) were removed and immediately immersed in 1 mL of RNAlater™ solution (Invitrogen, Thermo Fisher Scientific, Loughborough, UK), stored at 4 °C for 24 h, and then moved to −80 °C for long-term storage until RNA extraction.

The remaining 3 fish per replicate (9 per treatment) were dissected under aseptic conditions to collect digesta from the PI for 16S rRNA gut microbiome analysis. Samples were placed in sterile, nucleic acid-free microcentrifuge tubes and stored at −80 °C until DNA extraction.

### 2.4. Growth Performance

After the 9-week feeding period, the final biomass of fish in each tank was weighed to assess growth performance using the following parameters:

$$\mathrm{Mean}\;\mathrm{weight}\;\mathrm{gain}\;\left(g\right)=\mathrm{Final}\;\mathrm{body}\;\mathrm{weight}\;\left(g\right)-\mathrm{Initial}\;\mathrm{body}\;\mathrm{weight}\;\left(g\right)$$

$$\mathrm{Specific}\;\mathrm{growth}\;\mathrm{rate}\;\left(\%\;/\mathrm{day}\right)=\frac{\left(ln\;\mathrm{Final}\;\mathrm{weight}\;\left(g\right)-ln\;\mathrm{Initial}\;\mathrm{weight}\;\left(g\right)\right)}{\mathrm{Days}\;\mathrm{fed}}\times 100$$

$$Feed\;conversion\;ratio=\frac{Feed\;intake\left(g\right)}{Weight\;gain\left(g\right)}$$

$$Fulton’s\;condition\;factor=\frac{Weight*100}{{Length}^{3}}$$

### 2.5. Histology

#### 2.5.1. Light Microscopy

The fixed skin (*n* = 9 per treatment) and PI (*n* = 9 per treatment) samples were dehydrated with a Leica TP 1020 tissue processor (Leica Biosystems, Sheffield, UK) and embedded in paraffin wax using a Leica EG1150 H (Leica Biosystems, Sheffield, UK). Multiple transverse sections of 5 μm were then prepared on slides with a Leica RM2235 microtome (Leica Biosystems, Sheffield, UK) and stained with H&E (Haematoxylin and Eosin), AB/PAS (Alcian Blue/Periodic Acid Schiff), or Van Gieson using a Leica Autostainer XL (Leica Biosystems, Sheffield, UK). Photomicrographs of the stained sections were taken using a Leica DMD 108 microscope (Leica Biosystems, Sheffield, UK). The images were analysed for various parameters using ImageJ v1.54 software (National Institutes of Health, Bethesda, MD, USA). Morphometric measurements, including mucosal fold length, muscularis thickness, and lamina propria width, were taken from H&E-stained PI sections, with five folds measured per section for each parameter. Goblet cell counts in skin and PI samples were assessed using AB/PAS- and Van Gieson-stained sections. For PI samples, goblet cells were counted across five folds per image and averaged over a standardised distance of 100 μm per fold. For skin samples, counts were averaged over five separate 200 μm standardised distances per section.

#### 2.5.2. Electron Microscopy

The fixative (2.5% glutaraldehyde) in the PI samples for SEM (*n* = 9 per treatment) and TEM (*n* = 9 per treatment) was replaced twice with 0.1 M sodium cacodylate buffer (pH = 7.2) for 15 min each to ensure complete removal. SEM samples were then dehydrated through a series of incremental ethanol solutions up to 100% for 15 min each. The samples were then transferred into a 2:1 solution of 100% ethanol and hexamethyldisilazane (HMDS) for 20 min, followed by a 1:2 100% ethanol–HMDS solution for another 20 min, and then placed in fresh 100% HMDS twice for 20 min each. Afterward, they were left in HMDS overnight to dry. The dried samples were affixed to stubs using silver paint, coated with gold, and imaged with a JEOL JSM-6610LV Scanning Electron Microscope (JEOL, Tokyo, Japan) at ×20,000 magnification.

Samples for TEM were post-fixed in 1% OsO_4_ for 1 h, and then rinsed with sodium cacodylate 0.1 M buffer solution. The samples were then dehydrated through incremental ethanol concentrations before being embedded in resin following the standard procedure. Ultrathin sections (90 nm) were then cut from the resin blocks with a diamond knife, mounted on copper grids, and stained with uranyl acetate and Reynolds lead citrate. Micrographs of microvilli length were captured at ×20,000 magnification using a JEOL 1400 Transmission Electron Microscope (JEOL, Tokyo, Japan) at 120 kV.

All images were analysed with ImageJ v1.54 software. SEM images were analysed to quantify the microvilli density (arbitrary units [AU]), while the TEM images were analysed to measure the microvilli length, as outlined by Merrifield et al. [20].

### 2.6. Haematology

#### 2.6.1. Haemoglobin Concentration

The haemoglobin concentration was determined using the Drabkin’s reagent (Sigma-Aldrich, Poole, UK) protocol. The blood samples (*n* = 9 per treatment) mixed with the Drabkin’s solution were gently homogenised and left at room temperature for 5 min before placing in a spectrophotometer to read the absorbance at 540 nm. The haemoglobin concentrations in the test samples were determined from a standard curve of lyophilised haemoglobin porcine powder (Sigma-Aldrich, Poole, UK) and calculated using the following formula:

$$Haemoglobin\;concentration\;(g/dL)=\frac{A_{540}\;of\;sample\times Dilution\;factor}{A_{540}\;of\;standard}$$

where A_540_ = absorbance at 540 nm.

#### 2.6.2. Blood Cell Count

The red blood cell (RBC) and white blood cell (WBC) counts were determined using the Dacie’s solution protocol. The mixed blood and Dacie’s solution (*n* = 9 per treatment) was vortexed for 60 s to ensure adequate dispersion of the blood cells. Ten μL of the sample was then pipetted onto a Neubauer haemocytometer (LO−LaborOptik, Lancing, UK to estimate the total RBC and WBC following standard protocol.

### 2.7. Gene Expression

#### 2.7.1. RNA Extraction and cDNA Synthesis

RNA extraction from the PI and cDNA synthesis were performed following the procedures described by Rawling et al. [21]. Briefly, for total RNA extraction, samples (*n* = 9 per treatment) were placed in lysing tubes containing 1 mL of TRI reagent™ solution (Invitrogen, Thermo Fisher Scientific, Loughborough, UK) and homogenised using the FastPrep-24™ 5G lysis system (MP Biomedicals, Irvine, CA, USA). Phase separation was achieved by adding chloroform, followed by RNA precipitation with isopropanol. The resulting RNA pellets were washed with ethanol and dissolved in DEPC-treated water. The Qiagen RNeasy Plus Mini Kit (QIAGEN, Manchester, UK) was subsequently used to further purify the RNA and remove any DNA contamination. The quality and concentration of RNA were assessed by measuring absorbance ratios at 260/230 nm and 260/280 nm using the NanoDrop™ 2000 UV-Vis spectrophotometer (Thermo Fisher Scientific, Loughborough, UK). RNA purity and integrity were further confirmed using agarose gel electrophoresis.

For cDNA synthesis, 1 μg of extracted RNA was used in a 20 μL reaction volume with the iScript cDNA synthesis kit (Bio-Rad, Watford, UK). The reaction consisted of a priming step (5 min at 25 °C), followed by reverse transcription (20 min at 46 °C), and a final heat inactivation step (95 °C for 1 min).

#### 2.7.2. Quantitative Real-Time PCR (qPCR)

The qPCR reaction for each sample was carried out using the SYBR green method and performed on a QuantStudio 12K Flex Real-Time PCR System (Applied Biosystems, USA), following the protocol outlined by Rawling et al. [21]. The relative expression level of 5 target genes, interleukin-1 beta (*il-1β*), tumor necrosis factor alpha (*tnf-α*), interleukin-10 (*il-10*), mucin 2 (*muc-2*), and claudin 15 (*cldn-15*), were analysed. Two reference genes, glyceraldehyde 3-phosphate dehydrogenase (*gapdh*) and elongation factor 1 alpha (*ef1α*), were used to normalise the results for each sample. Prior to the qPCR analysis of each sample, the expression stability measure “M” for the reference genes was determined [22]. All primers used for this gene expression analysis (Table 3) were from a previous study [23]. The primers were further validated by conducting a PCR efficiency analysis using the method of Rasmussen [24].

### 2.8. Intestinal Microbiome Analysis

#### 2.8.1. DNA Extraction

DNA extraction from digesta samples (*n* = 9 per treatment) was conducted using the QIAamp PowerFecal Pro DNA Kit (QIAGEN, Manchester, UK) in accordance with the manufacturer’s protocol. Briefly, 250 mg of digesta was placed in a lysis tube with 800 µL of lysis buffer and vortexed for 10 min to ensure homogenisation. After centrifuging the mixture, the supernatant was treated with precipitation buffers to isolate the DNA, which was further purified through a series of wash steps in a spin column. The purified DNA was eluted in 50 µL of elution buffer and assessed for quality and concentration using a NanoDrop™ 2000 UV-vis spectrophotometer (Thermo Fisher Scientific, Loughborough, UK). The DNA samples were stored at −20 °C for downstream applications.

#### 2.8.2. PCR Amplification and High-Throughput Sequencing

The PCR amplification and sequencing were conducted by Novogene Company Limited, Cambridge, UK. The 16S V3-V4 region was amplified using the primers 341F (5′-CCTACGGGNGGCWGCAG-3′) and 805R (5′-GGACTACNNGGGTATCTAAT-3′). Each PCR reaction contained approximately 10 ng of template DNA, 0.2 µM primers, and 15 µL Phusion High-Fidelity PCR Master Mix. Thermal cycling conditions included an initial denaturation at 98° C for 1 min, followed by 30 cycles of denaturation at 98 °C for 10 s, annealing at 50 °C for 30 s, and elongation at 72 °C for 30 s, with a final elongation at 72 °C for 5 min. The PCR products were purified with magnetic beads, pooled in equimolar concentrations, and then prepared for sequencing by adding indexed adapters. The libraries were quantified and validated before sequencing on an Illumina platform to provide high-resolution microbial community data.

The sequencing data were processed by merging paired-end reads using FLASH software v1.2.11 [25], and then filtered for quality with fastp [26]. Chimeric sequences were identified and removed using VSEARCH v2.16.0 [27]. High-quality sequences were then denoised with DADA2 to generate Amplicon Sequence Variants (ASVs) [28], which were taxonomically classified against the SILVA 138.1 database using the QIIME2 pipeline v202202 [29]. Phylogenetic relationships were analysed using QIIME2, and diversity metrics (alpha and beta) were computed based on normalised sequence data to examine community structure variations. UPGMA clustering was employed for dimensionality reduction and visualisation of community differences across samples, enabling detailed insights into microbial community composition.

### 2.9. Statistical Analysis

The statistical analysis of qPCR data was conducted using RStudio v2024.04.0+735 (RStudio PBC, Boston, MA, USA), while growth performance, haematology, and histology data were analysed using SPSS v22 (SPSS Inc., Chicago, IL, USA). For RT-qPCR data, a permutation test was applied following Röhmel [30]. Growth, haematology, and histology data were first assessed for normality using the Shapiro–Wilk test. Normally distributed data were analysed with one-way ANOVA, followed by Tukey’s HSD *post hoc* test to identify significant differences between treatment groups. For non-normally distributed data, the Kruskal–Wallis test was applied, with Dunn’s post hoc test for pairwise comparisons.

Intestinal microbiota sequencing data were analysed using R software v4.0.3 with vegan (v2.6-8) and ggplot2 (v3.5.1) packages. To assess differences in species composition and community structure, several statistical tests were performed, including Analysis of Similarity (ANOSIM), Permutational Multivariate Analysis of Variance (ADONIS), and ANOVA. Significance was accepted at *p* < 0.05 for all tests.

## 3. Results

### 3.1. Histology

#### 3.1.1. Light Microscopy

**Figure 1:**
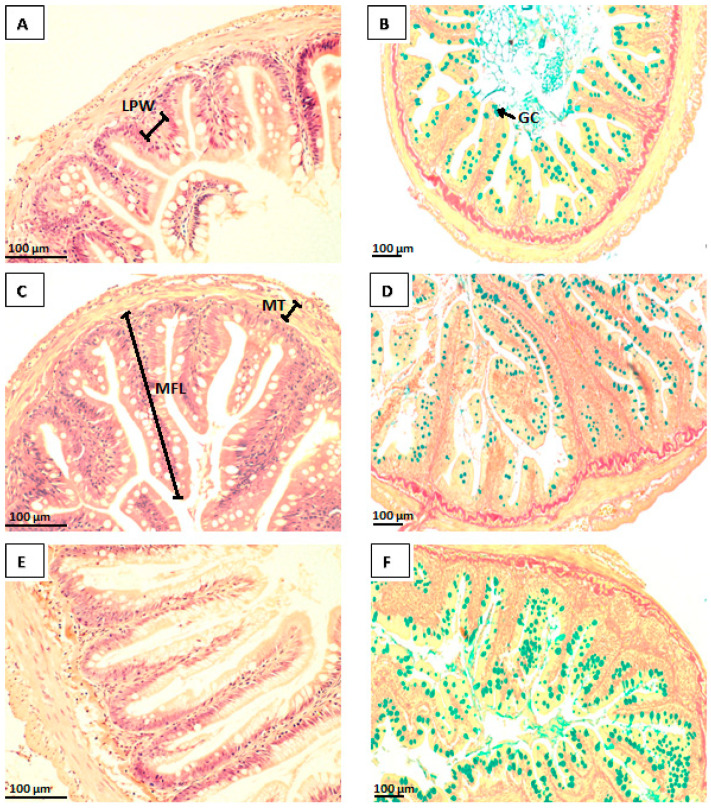
Photomicrographs of the posterior intestine of Atlantic salmon parr fed control (**A**,**B**), ABY (**C**,**D**), and SDYE (**E**,**F**) diets. Images (**A**,**C**,**E**) depict cross-sections stained with H&E, and images (**B**,**D**,**F**) show cross-sections stained with Van Gieson (scale bars = 100 μm). LPW: lamina propria width, MFL: mucosal fold length, MT: muscularis thickness, and GC: goblet cells.

**Figure 2:**
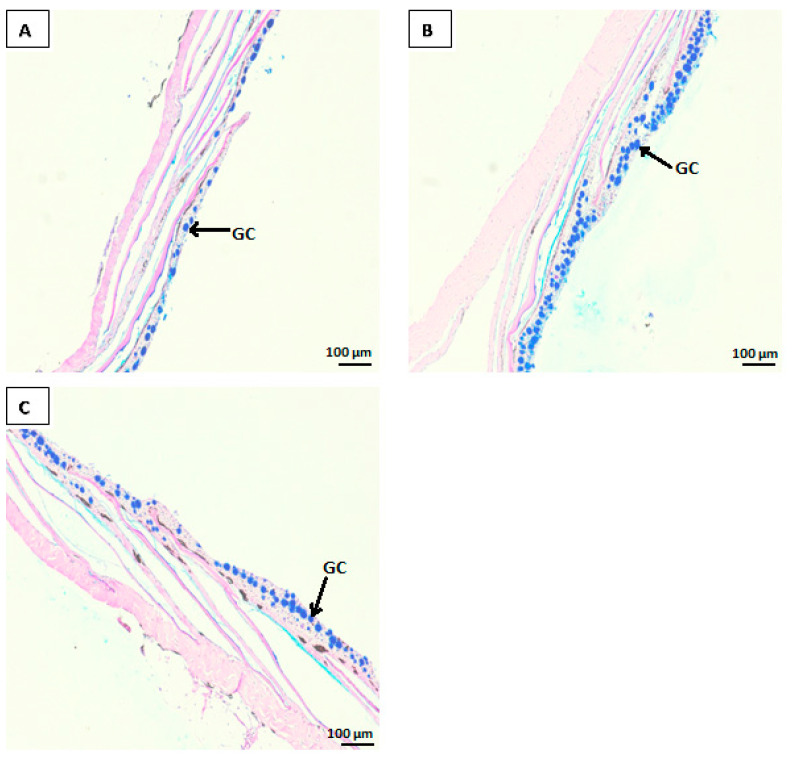
Photomicrographs of AB/PAS-stained skin tissues from Atlantic salmon parr fed control (**A**), ABY (**B**), and SDYE (**C**) diets, with goblet cell indicated (scale bars = 100 μm). GC: goblet cells.

The light microscopy histological analysis results of the PI and skin of Atlantic salmon parr are presented in Table 4. The photomicrographs revealed that fish from all treatment groups exhibited an intact epithelial barrier and well-organised mucosal folds, consisting of a simple lamina propria and numerous goblet cells (Figure 1 and Figure 2). The results showed that fish fed yeast-supplemented diets, especially the ABY treatment group, had improved intestinal morphology, including a higher number of goblet cells in both the intestine and skin. Specifically, the mucosal fold length was significantly longer (*p* < 0.05) in the ABY (307.41 ± 66.03 µm) and SDYE (375.98 ± 69.77 µm) groups compared with the control group (203.74 ± 35.04 µm). Additionally, the lamina propria width in fish fed the ABY diet (11.86 ± 2.11 µm) was significantly narrower (*p* = 0.04) compared with those fed the control diet (16.81 ± 6.13 µm). The number of goblet cells in the PI of fish in the ABY group (9.44 ± 1.91 n/100 µm) was significantly higher (*p* = 0.04) than in the control group (7.01 ± 1.71 n/100 µm). Similarly, the goblet cell counts in the skin of fish receiving the ABY diet (18.22 ± 2.48 n/200 µm) were significantly higher (*p* = 0.02) than those of the control group (14.56 ± 2.85 n/200 µm).

#### 3.1.2. Electron Microscopy

**Figure 3:**
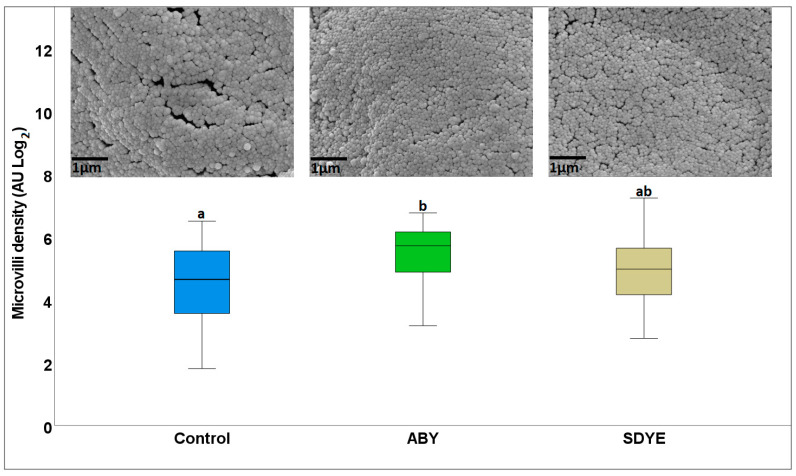
Microvilli density of the Atlantic salmon parr posterior intestine after the 9-week experimental feeding. AU = arbitrary units. Different superscript letters for individual parameters among dietary groups indicate significant differences (*p* < 0.05).

**Figure 4:**
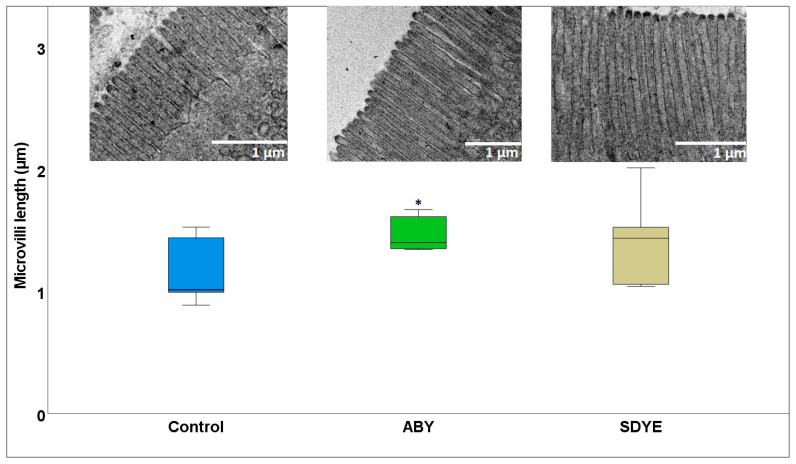
Microvilli length of the Atlantic salmon parr posterior intestine after the 9-week experimental feeding. * *p* = 0.052 between ABY and control.

The electron microscopy result revealed a healthy brush border with densely packed microvilli across all treatment groups, with no evidence of necrosis or structural disruption (Figure 3 and Figure 4). The microvilli density in Atlantic salmon parr fed the ABY diet (5.36 ± 1.12 AU) was significantly higher (*p* = 0.03) than in those fed the control diet (4.57 ± 1.27 AU) (Figure 3). Similarly, the microvilli length (1.46 ± 0.14 µm) in the fish fed ABY were numerically longer than in the control group (1.15 ± 0.27 µm), with a *p* value approaching significance (*p* = 0.052) (Figure 4).

### 3.2. Haematology

The haemoglobin concentration and blood cell counts in Atlantic salmon parr fed the experimental diets for the 9-week feeding period are shown in Table 5. The Atlantic salmon in all treatment groups exhibited normal blood parameters. The haemoglobin concentration, RBC, and WBC counts were not significantly different (*p* > 0.05) among the treatment groups.

### 3.3. Gene Expression

**Figure 5:**
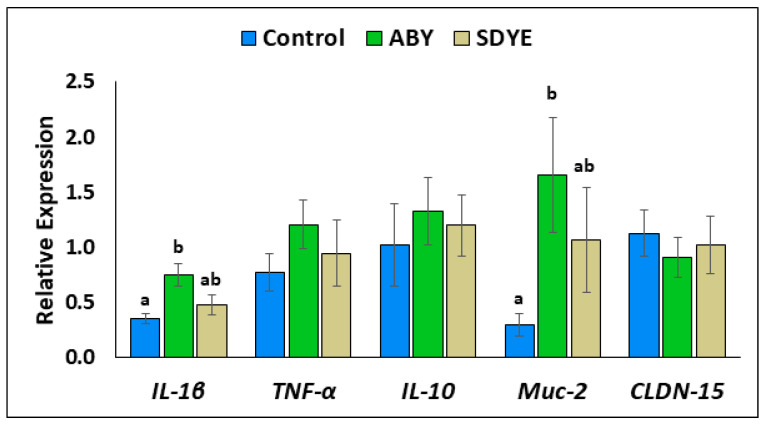
Relative gene expression profiles in the posterior intestine of Atlantic salmon parr after the 9-week feeding trial. Different superscript letters denote significant differences between treatments (*p* < 0.05). Data presented as mean ± standard error of the mean (SEM).

The relative expressions of the five target genes in the PI of Atlantic salmon parr following the 9-week feeding trial are presented in Figure 5. The result showed the expression level of *il-1β* in the PI of Atlantic salmon parr fed the ABY diet (0.88 ± 0.12) to be significantly higher (*p* = 0.004) than that in the control group (0.41 ± 0.05). Similarly, *muc-2* expression was significantly upregulated (*p* = 0.01) in Atlantic salmon fed the ABY diet (1.94 ± 0.61) compared with in those fed the control diet (0.35 ± 0.11). All other analysed genes (*tnf-α*, *il-10*, and *cldn-15*) exhibited no significant difference (*p* > 0.05) across the treatment groups.

### 3.4. Intestinal Microbiome

#### 3.4.1. Sequencing and ASVs Identification

**Figure 6:**
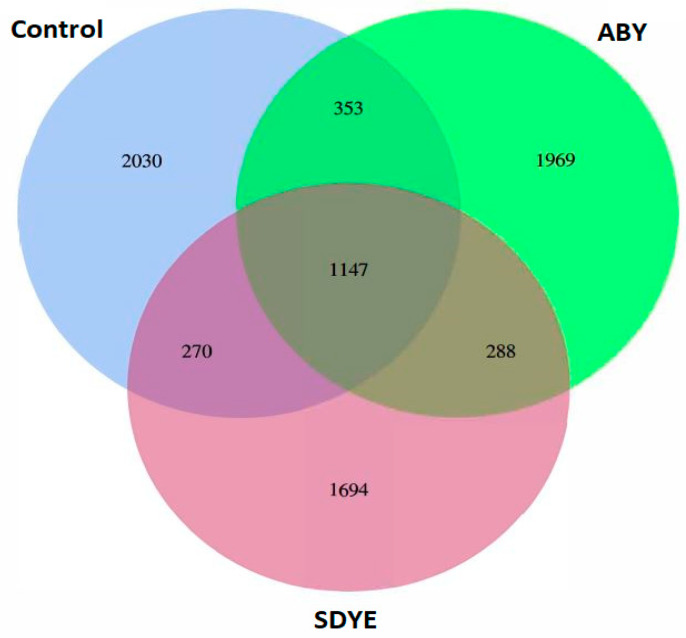
Shared and unique ASVs in the digesta samples of Atlantic salmon parr across treatment groups.

A total of 2,851,529 raw paired-end reads were obtained across all treatment groups. Following quality control and removal of chimeras, 2,610,365 high-quality reads were retained for downstream analysis, with mean values of 100,899 ± 6,575 for the control, 94,188 ± 2,059 for ABY, and 94,952 ± 5,462 for SDYE (*n* = 9 per treatment) (Table 6). From these 27 samples, 7,751 unique ASVs were identified, representing a total of 2,456,278 sequences (Figure 6). Among the detected ASVs, 14.8% (1,147 ASVs) were common to all the three treatment groups. Meanwhile, 26.2% (2,030 ASVs) were specific to the control group, 25.4% (1,969 ASVs) were unique to the ABY group, and 21.9% (1,694 ASVs) were unique to the SDYE group.

#### 3.4.2. Diversity and Community Structure

**Figure 7:**
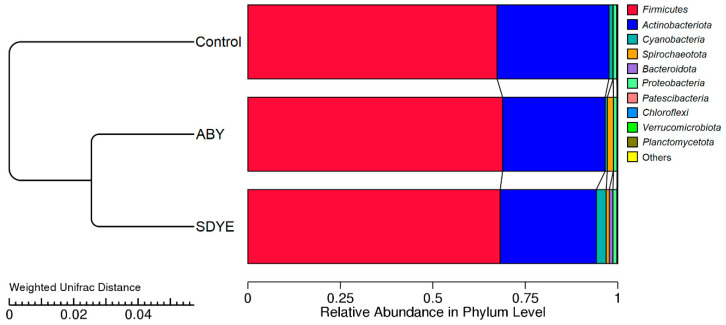
UPGMA cluster tree based on weighted UniFrac distance for classifying the most abundant phyla in the digesta samples of Atlantic salmon parr across the three treatment groups.

Good’s coverage values of 1 were achieved for all treatment groups, indicating that the sequencing depth was sufficient to accurately capture the full diversity of the ASVs present in the sampled populations. The alpha diversity indices revealed no significant differences (*p* > 0.05) between the treatment groups (Table 7). However, UPGMA cluster analysis using weighted UniFrac distances (Figure 7) revealed that the communities of the two yeast treatment groups were more similar to one another than they were to those of the control group. ANOSIM values (Table 8) indicated moderate (R = 0.42816; *p* = 0.001) or weak (R = 0.19342; *p* = 0.002), yet statistically significant, separation of ABY and SDYE communities compared with the control group communities, respectively. ADONIS results (Table 8) indicated the same with F = 4.311 and F = 3.125 for control–ABY and control–SDYE, respectively (*p* < 0.002).

#### 3.4.3. Bacterial Composition

**Figure 8:**
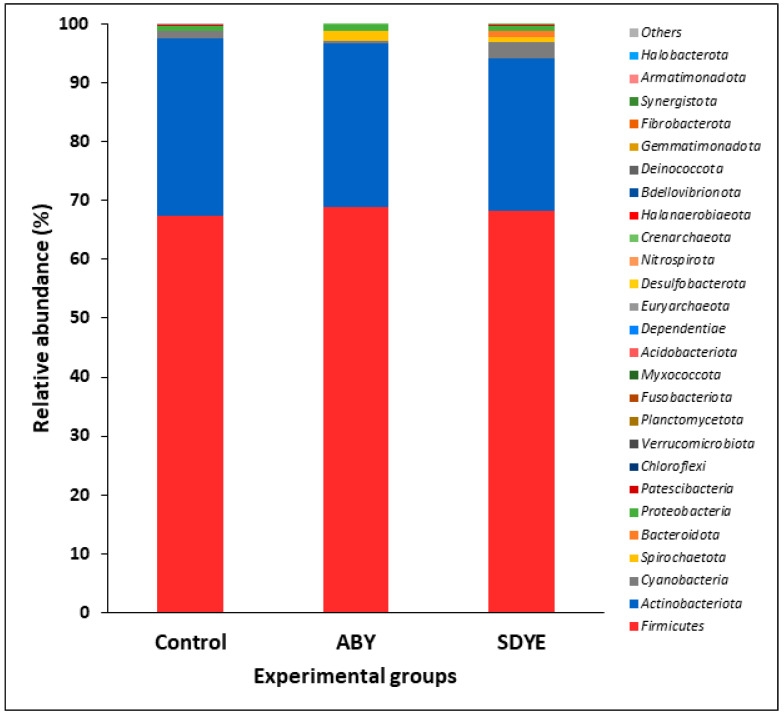
Relative abundance of the 26 bacterial phyla detected in the digesta of Atlantic salmon parr in each treatment group.

**Figure 9:**
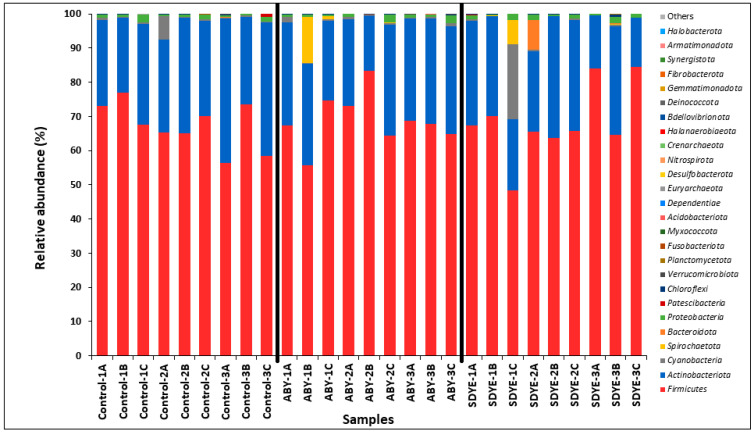
Relative abundance of the 26 bacterial phyla detected in the 27 individual digesta samples.

The digesta of the Atlantic salmon parr across all treatment groups contained 26 bacterial phyla, representing over 99.9% of the annotated sequences (Figure 8 and Figure 9). *Firmicutes* was the dominant and most prevalent phylum across all three treatment groups, comprising approximately 68% of ASVs in each group. *Actinobacteriota* followed as the second most abundant phylum, accounting for 26% of ASVs in the SDYE group, 28% in ABY, and 30% in the control group. The remaining 24 phyla collectively contributed 2–6% of the annotated sequences across all groups, with notable phyla including *Cyanobacteria*, *Proteobacteria*, *Spirochaetota*, *Bacteroidota*, *Patescibacteria*, *Chloroflexi*, *Verrucomicrobiota*, *Planctomycetota*, *Fusobacteriota*, and *Myxococcota*.

**Figure 10:**
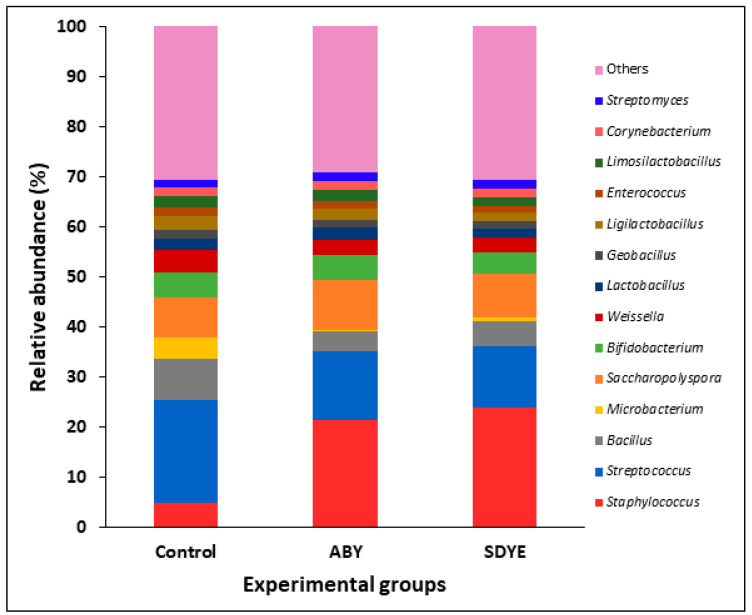
Relative abundance of the top 14 dominant genera observed in digesta of the Atlantic salmon parr across treatment groups.

**Figure 11:**
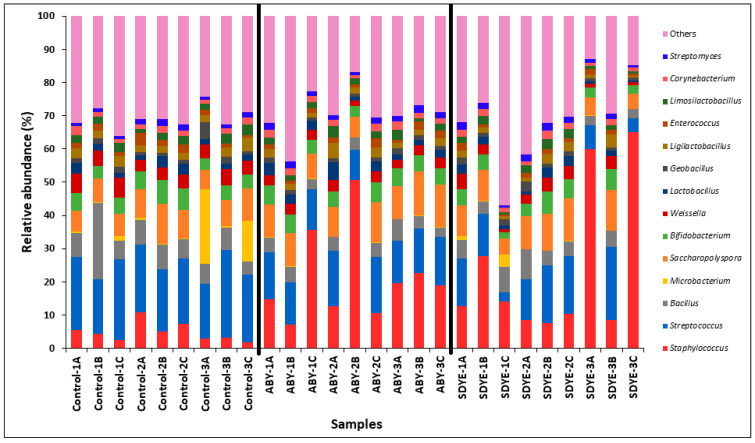
Relative abundance of the top 14 dominant genera observed in the 27 individual digesta samples.

**Figure 12:**
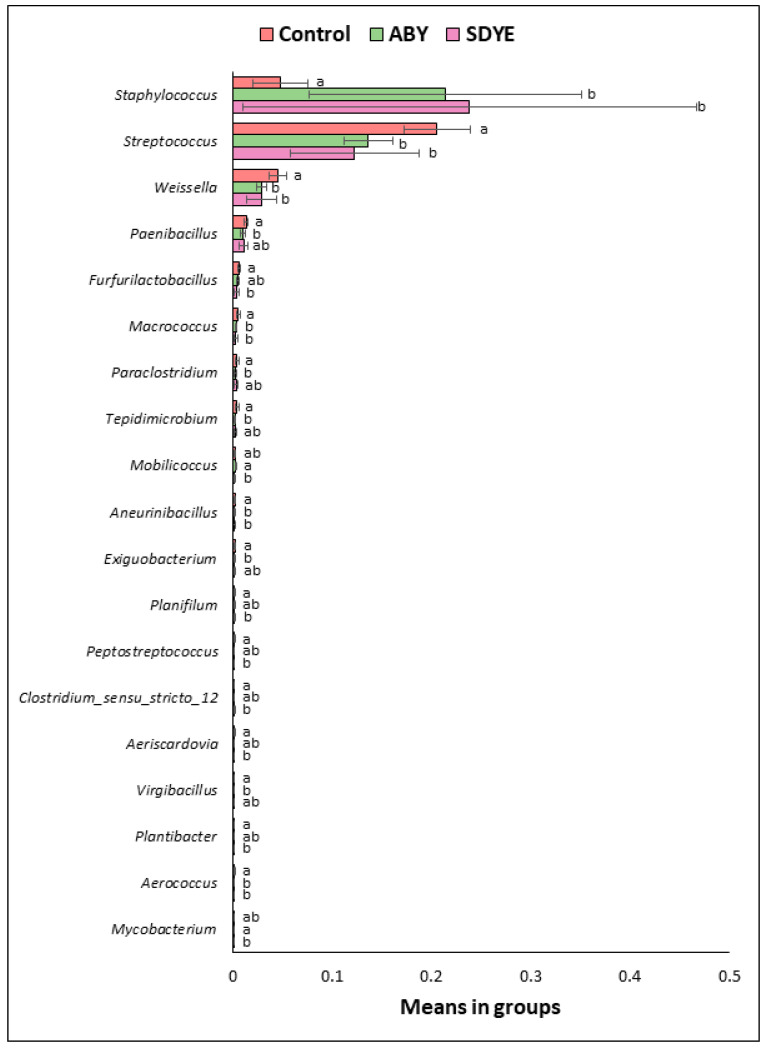
Statistical differences in bacteria genera with mean relative abundance ≥ 0.1% across the three treatment groups. Different superscript letters denote significant differences between treatments (*p* < 0.05). Data presented as mean ± standard deviation (SD).

At the genus level, 597 bacterial taxa were classified from 2,286,589 sequences. *Staphylococcus* and *Streptococcus* were the most prevalent bacteria in the digesta of the Atlantic salmon across all treatments (Figure 10 and Figure 11). Other dominant bacterial genera identified include *Saccharopolyspora*, *Bacillus*, *Bifidobacterium*, *Weissella*, *Ligilactobacillus*, *Lactobacillus*, *Limosilactobacillus*, *Corynebacterium*, *Microbacterium*, *Streptomyces*, *Enterococcus*, and *Geobacillus*. Significant differences (*p* < 0.05) were observed in the relative abundance of certain genera between the control and other treatment groups (Figure 12). Notably, *Staphylococcus* abundance was significantly higher (*p* < 0.05) in fish fed ABY and SDYE diets compared with the control diet. In contrast, *Streptococcus*, *Weissella*, *Macrococcus*, and *Aerococcus* were significantly more abundant (*p* < 0.05) in the control-fed fish compared with the ABY and SDYE groups.

### 3.5. Growth Performance

The growth performance of the Atlantic salmon fed the experimental diets for 9 weeks is presented in Table 9. Atlantic salmon parr exhibited 100% survival across all treatment groups. Growth parameters such as final body weight, mean weight gain, specific growth rate, and feed conversion ratio showed no significant differences (*p* > 0.05) between treatments. The Fulton’s condition factor for all treatment groups was also not significantly different (*p* > 0.05).

## 4. Discussion

This study evaluated the effects of autolysed brewer’s yeast (ABY) and soluble dried yeast extract (SDYE) dietary supplementation at 2.5 g/kg on the intestinal and skin histology, haematology, immunoregulatory gene expression, microbiota composition, and growth performance of Atlantic salmon parr. The findings demonstrate that fish receiving yeast-supplemented diets, especially the ABY diet, exhibited enhanced mucosal health, modulated immune gene expression, and distinct gut microbiome profiles, while growth performance remained consistent across treatments. These results support the potential of yeast-based additives in aquaculture, particularly in enhancing fish health without negatively impacting growth.

Histological analysis revealed that Atlantic salmon fed the ABY diet exhibited increased mucosal fold length, narrower lamina propria, higher microvilli density, longer microvilli length, and enhanced goblet cell counts in the intestine and skin compared with the control group. These histological changes indicate that the ABY additive positively impacts intestinal morphology, promoting gut health. Longer mucosal folds and increased goblet cell numbers are associated with enhanced nutrient absorption and mucosal immunity, essential for maintaining intestinal health and resisting pathogens [31,32]. Similar to the findings of this study, Leclercq et al. [14] reported enhanced skin mucus and goblet cell density along with an increased intestinal goblet cell density and villi length in Atlantic salmon fed a diet supplemented with commercial yeast MOS at 4 g/kg for 65 days. Adeoye et al. [33] also reported increased intestinal goblet cell counts in African catfish (*Clarias gariepinus*) fed diets supplemented with ABY at 3 to 10 g/kg levels. The histological improvements in the ABY group may be attributed to bioactive components within the autolysed brewer’s yeast, such as β-glucans and MOS, which have been shown to promote mucosal immunity and gut health [34,35]. These components, found in yeast cell walls, stimulate immune responses, and promote intestinal integrity by enhancing cellular structure [32]. In contrast, the SDYE lacks these cell wall components, potentially explaining its relatively modest impact on histology compared with the ABY additive. However, SDYE still conferred improved mucosal morphology compared with controls, indicating that yeast extracts (nucleotides, peptides) also contribute to gut health [36].

Haematological parameters, including haemoglobin concentration, RBC, and WBC, are essential indicators for assessing fish health, physiological responses, and adaptation to environmental or dietary changes [37,38]. Our results showed that the blood profiles of the Atlantic salmon after 9 weeks of experimental feeding were stable, with no significant changes observed in the parameters across treatment groups. This suggests that ABY and SDYE dietary supplementation at 2.5 g/kg level did not compromise the Atlantic salmon health. This outcome is consistent with other studies that reported no significant haematological changes in fish fed diets supplemented with yeast additives. For example, Kühlwein et al. [38] reported that supplementation of β-glucan at 1 g/kg, 10 g/kg, and 20 g/kg in the diet of mirror carp (*Cyprinus carpio*) did not have any detrimental effect on the haematological parameters. Similarly, Neuls et al. [39] reported safe inclusion of *Yarrowia lipolytica* yeast in the diet of Nile tilapia (*Oreochromis niloticus*) as no significant changes were observed in the blood parameters when compared with the control.

The gene expression analysis in this study showed the potential of the yeast additives, especially ABY, in modulating some immunoregulatory genes in the PI of the Atlantic salmon parr. The *il-1β* and *muc-2* genes were significantly upregulated in Atlantic salmon fed the ABY diet, indicating enhanced inflammatory and mucosal responses. The proinflammatory cytokine *il-1β*, crucial for early immune responses, plays a key role in initiating the immune cascade and improving pathogen resistance [40]. The *muc-2* gene is a key mucin protein responsible for forming protective mucus barriers that protects the intestinal lining [41]. The increased expression of *il-1β* and *muc-2* in the ABY group aligns with previous findings suggesting that yeast additives enhance mucosal protection and activate immune responses without inducing systemic inflammation. A significant upregulation of *il-1β* in the mid-intestine was previously reported in Nile tilapia fed a diet containing ABY at a dose of 1 g/kg for 5 weeks [7]. Similarly, Andriamialinirina et al. [42] reported that *il-1β* and other cytokines were significantly elevated in the intestine of Nile tilapia fed a diet supplemented with brewer’s yeast hydrolysate at a level of 1 g/kg for 8 weeks. Furthermore, Huang et al. [43] reported that concentrated MOS supplemented at 4 g/kg in the diet of goldfish (*Carassius auratus*) for 60 days modulated intestinal gene expression by increasing *il-1β* and *muc-2* expression.

The significant upregulation of the *muc-2* gene in the PI of Atlantic salmon fed ABY-supplemented diets in this study is consistent with the significantly increased number of goblet cells observed in the same group of fish. Goblet cells secrete mucins such as *muc-2*, which are glycoproteins with gelling properties that provides the viscous and adhesive properties of mucus, supporting gut integrity and protecting against pathogens [44].

The gut microbiota is vital to animal health, including in fish, as it supports nutrient absorption, immune function, and overall well-being [45]. The alpha diversity analyses revealed no significant differences between treatments, suggesting a similarly diverse microbial community across groups. However, UPGMA cluster analysis and community structure tests (ANOSIM and ADONIS) highlighted distinct compositional shifts in the microbiome between control and yeast-supplemented diets, underscoring the capacity of yeast additives to modulate gut microbiota. Similar findings in gilthead seabream and hybrid striped bass support the notion that dietary yeast additives can influence microbial composition [46,47].

The gut microbiome analysis indicated that *Firmicutes* and *Actinobacteriota* were predominant across treatments, which aligns with previous research in Atlantic salmon and other fish species [48,49]. Meziti et al. [49] identified *Firmicutes* and *Actinobacteriota* as dominant phyla in the gut and faecal samples of European seabass (*Dicentrarchus labrax*) fed yeast-supplemented diets. Agboola et al. [48] also reported these two phyla to be among the top three most abundant phyla in the digesta of Atlantic salmon fed *Cyberlindnera jadinii* and *Wickerhamomyces anomalus* yeast additives. These bacterial phyla include members such as *Lactobacillus*, *Bacillus*, *Enterococcus*, and *Streptomyces*, which are essential for supporting intestinal health and enhancing immune function in fish [50].

The gut microbiome result revealed some overlap in the microbial communities, but yeast supplementation notably influenced the relative abundance of specific bacterial taxa in the fish digesta. Specifically, the relative abundance of *Staphylococcus* increased in the digesta of fish fed yeast-supplemented diets, while *Streptococcus* and *Weissella* were more abundant in the control group. *Staphylococcus* comprises Gram-positive anaerobic bacteria with a chemoorganotrophic metabolism, utilizing carbohydrates and amino acids as carbon and energy sources through respiratory and fermentative pathways [51]. This genus, though infrequently encountered and studied in fish microbiomes [52], has been associated with dietary influences and observed in the gut microbiota of fish fed soy proteins and vegetable oil-based diets [49,53]. This could explain the dominance of this genus in this study as the diets were formulated with relatively high amounts of soybean meal and vegetable oil. Human studies indicate that live yeast can aid adhesion of staphylococci to host cells and tissues, partially through biofilm formation [54], but it is not clear in our study if the yeast additives influenced the adhesive capacity of the *Staphylococcus*. Similar microbiome shifts with *Staphylococcus* enrichment were reported in a study on European seabass, where it was found to be the most abundant genus in the intestine of European seabass fed diets formulated with soy proteins and yeast additives [49]. Pérez-Pascual et al. [55] also reported increased *Staphylococcus* abundance in the gut of European seabass fed diets supplemented with yeast additives. In addition, Burr et al. [46] reported a similar increase in *Staphylococcus* in the intestine of hybrid striped bass (*Morone chrysops* × *Morone saxatilis*) fed diets supplemented with different yeast additives such as MOS and partially autolysed brewer’s yeast. 

Conversely, the reduction of *Streptococcus* and *Weissella* in the ABY and SDYE groups may be attributed to the prebiotic and immune-modulating effects of yeast. Yeast additives may modulate gut microbial composition by supporting beneficial bacterial growth and reducing potentially harmful taxa. Such shifts align with research showing yeast additives can promote microbial diversity and balance [49,53]. This decrease could indicate a decline in potentially opportunistic bacteria, as some *Streptococcus* species are known to be pathogenic in fish, causing streptococcosis [56]. Although most *Weissella* spp. have been reported to be beneficial and have probiotic effect on fish [57], some strains such as *Weissella ceti* have been recognised to act opportunistically and cause infections [58,59]. Prior studies have also reported a similar decrease in the abundance of *Streptococcus* and *Weissella* in the fish gut. For example, Meziti et al. [49] reported significant reduction in the abundance of *Streptococcus* in the gut of European sea bass fed yeast-supplemented diets.

## 5. Conclusions

Overall, the study demonstrates that dietary supplementation with autolysed brewer’s yeast and soluble dried yeast extract can positively impact intestinal morphology, mucosal immunity, and microbial composition in Atlantic salmon parr. The differential responses between ABY and SDYE treatments point to a potential advantage of yeast wall components in enhancing gut health, which could be valuable under diverse rearing or nutritional conditions. These findings significantly advance our understanding of how yeast additives can improve mucosal health in Atlantic salmon. However, further research is required to clarify the underlying mechanisms of these effects and to optimise the inclusion levels of ABY and SDYE in Atlantic salmon diets, ensuring growth and other benefits across different rearing conditions. It is also recommended that further research should include a gradient design with a varying inclusion level of the yeast additives to determine their optimal inclusion level for Atlantic salmon.

## Figures and Tables

**Table 1:** Nutritional composition of the test yeast additives (Leiber GmbH).

	CeFi^®^ Pro	SDYE
Crude protein	50.0%	65.0%
Crude fibre	1.0%	1.0%
Crude ash	6.6%	9.4%
Crude fat	3.0%	0.1%
Moisture	5.0%	5.0%

**Table 2:** Formulations and proximate compositions of the experimental diets.

Ingredients (g/kg)	Control	ABY	SDYE
Soybean meal 48 ^1^	150.0	150.0	150.0
Sunflower meal ^2^	159.4	157.8	157.8
Fishmeal ^2^	70.0	70.0	70.0
Corn gluten meal ^2^	220.0	220.0	220.0
Wheat gluten meal ^2^	138.2	138.2	138.2
Fish oil ^2^	90.0	90.0	90.0
Sunflower oil ^3^	120.8	120.8	120.8
Lysine ^4^	20.0	20.0	20.0
DL methionine ^4^	3.2	3.2	3.2
Threonine ^4^	1.7	1.7	1.7
Histidine ^4^	3.1	3.1	3.1
Arginine ^4^	13.6	13.6	13.6
CMC binder ^4^	5.0	5.0	5.0
Vitamin and mineral premix ^5^	5.0	5.0	5.0
ABY ^6^	-	2.5	-
SDYE ^6^	-	-	2.5
**Proximate composition (% wet weight basis)**			
Protein	49.45 ± 0.16	48.73 ± 0.76	49.34 ± 0.07
Lipid	24.15 ± 0.07	23.39 ± 0.62	22.43 ± 1.32
Moisture	3.78 ± 0.10	3.93 ± 0.08	3.85 ± 0.13
Ash	4.46 ± 0.27	4.13 ± 0.15	4.28 ± 0.22
Nitrogen-free extract	18.16 ± 0.27	19.82 ± 0.45	20.10 ± 1.34
Gross energy (kcal/g)	5.82 ± 0.01	5.78 ± 0.03	5.73 ± 0.07

Proximate composition data presented as mean ± standard deviation (SD). ABY = autolysed brewer’s yeast; SDYE = soluble dried yeast extract. ^1^ Skretting, Stavanger, Norway. ^2^ Biomar, Aarhus, Denmark. ^3^ Tesco, Plymouth, UK. ^4^ Sigma-Aldrich, Poole, UK. ^5^ Premier nutrition vitamin/mineral premix (contains 121 g kg^−1^ of calcium, 5.2 g kg^−1^ of phosphorous, 15.6 g kg^−1^ of magnesium, 250 mg kg^−1^ of copper (as cupric sulphate), 7.0 g kg^−1^ of Vit E (as alpha-tocopherol acetate), 1.0 μg kg^−1^ of Vit A, 0.1 μg kg^−1^ of Vit D3, & 787 g kg^−1^ ash). ^6^ Leiber GmbH, Bramsche, Germany.

**Table 3:** Target and reference genes used for qPCR in the posterior intestine of Atlantic salmon, with their corresponding primer details.

Gene	Gene Annotation	NCBI Accession Number	Primer Sequence (5′–3′)
*gapdh*	Glyceraldehyde 3-phosphate dehydrogenase	XM_014141819.2	Fwd—GCACCCATCGCCAAGGTTAT Rev—AGTCTTCTGTGTGGCTGTGA
*ef1α*	Elongation factor 1 alpha	NM_001141909	Fwd—GGCTGATTGTGCTGTGCTTAT Rev—CACGAGTCTGCCCGTTCTTT
*il-1β*	Interleukin-1 beta	AY617117	Fwd—AGGAGGGAAGCAGGGTTCA Rev—CATCAGGACCCAGCACTTGT
*tnf-α*	Tumor necrosis factor alpha	NM_001123617.1	Fwd—GCACCGAAGACAACAAGGTTTA Rev—GCTGAACACTGCTCCCACATA
*il-10*	Interleukin-1 beta	EF165028.1	Fwd—ACGAAGGCATTCTACACCACTT Rev—CACCGTGTCGAGGTAGAACT
*muc-2*	Mucin 2-like	XM_029770456.1	Fwd—CGACTCAACGTGGATGTAGGA Rev—GCGACCACTAGCCAGAAAGA
*cldn-15*	Claudin 15	XM_014206890.1	Fwd—GTCGGGATGCAGTGTTCTAAAG Rev—TGGGTGATGTTGAAAGCATACC

**Table 4:** The posterior intestine and skin histology of the Atlantic salmon parr using light microscope after the 9-week experimental feeding.

Parameters	Control	ABY	SDYE
Mucosal fold length (µm)	203.74 ± 35.04 ^a^	307.41 ± 66.03 ^b^	375.98 ± 69.77 ^b^
Muscularis thickness (µm)	56.76 ± 13.73	72.97 ± 15.73	69.89 ± 13.11
Lamina propria width (µm)	16.81 ± 6.13 ^a^	11.86 ± 2.11 ^b^	13.31 ± 3.02 ^ab^
Intestine goblet cell counts (n/100 µm)	7.01 ± 1.71 ^a^	9.44 ± 1.91 ^b^	9.24 ± 2.44 ^ab^
Skin goblet cell counts (n/200 µm)	14.56 ± 2.85 ^a^	18.22 ± 2.48 ^b^	14.44 ± 2.66 ^a^

Different superscript letters for individual parameters among dietary groups indicate significant differences (*p* < 0.05). Data presented as mean ± standard deviation (SD).

**Table 5:** Haematological parameters in Atlantic salmon parr after the 9-week feeding trial.

Parameters	Control	ABY	SDYE
Haemoglobin (g/dL)	0.03 ± 0.01	0.04 ± 0.01	0.03 ± 0.01
Red blood cell count (10^6^/µL)	0.89 ± 0.34	1.19 ± 0.26	1.13 ± 0.20
White blood cell count (10^3^/µL)	11.32 ± 4.36	14.36 ± 3.63	12.71 ± 5.68

Data presented as mean ± standard deviation (SD).

**Table 6:** Raw paired-end reads, non-chimera reads, ASVs, and sequences assigned to ASVs of microbiota composition in Atlantic salmon digesta samples after the 9-week feeding trial.

Treatments	Raw Paired-End Reads	Non-Chimera Reads	Number of ASVs	Sequences Assigned to ASVs
Control	109,556 ± 6,279	100,899 ± 6,575	3,800	94,946 ± 6,458
ABY	104,255 ± 1,768	94,188 ± 2,059	3,757	88,385 ± 2,015
SDYE	103,025 ± 7,364	94,952 ± 5,462	3,399	89,588 ± 5,106

Data for raw paired-end reads, non-chimera reads, and sequences assigned to ASVs are presented as mean ± standard deviation (SD).

**Table 7:** Goods coverage and diversity/richness indices of the microbiota composition in Atlantic salmon digesta samples after the 9-week feeding trial.

Treatments	Good’s Coverage	Chao1 Index	Shannon’s Diversity Index	Simpson’s Index of Diversity
Control	1.00 ± 0.00	880.79 ± 182.64	6.94 ± 0.38	0.96 ± 0.01
ABY	1.00 ± 0.00	902.04 ± 80.44	6.85 ± 0.60	0.96 ± 0.03
SDYE	1.00 ± 0.00	775.58 ± 193.29	6.53 ± 1.08	0.94 ± 0.08

Data presented as mean ± standard deviation (SD).

**Table 8:** ANOSIM and ADONIS analyses of bacterial communities in digesta samples of Atlantic salmon after 9 weeks on experimental diets.

Groups	ANOSIM		ADONIS	
	R-Value	*p*-Value	F-Value	*p*-Value
Control–ABY	0.428	0.001	4.311	0.002
Control–SDYE	0.193	0.002	3.125	0.001
ABYE–SDYE	0.188	0.004	2.436	0.008

**Table 9:** Growth performance, feed utilisation, and survival of Atlantic salmon parr fed experimental diets for 9 weeks.

Parameters	Control	ABY	SDYE
Initial body weight (g)	37.10 ± 0.00	37.10 ± 0.17	37.03 ± 0.06
Final body weight (g)	66.64 ± 2.52	63.18 ± 1.58	67.69 ± 0.61
Mean weight gain (g)	29.54 ± 4.37	26.08 ± 2.58	30.65 ± 1.11
Feed intake (g)	27.42 ± 0.58	26.25 ± 0.90	27.45 ± 0.48
Specific growth rate (%/day)	0.93 ± 0.11	0.85 ± 0.06	0.96 ± 0.02
Feed conversion ratio	0.94 ± 0.12	1.01 ± 0.07	0.90 ± 0.02
Survival (%)	100.00 ± 0.00	100.00 ± 0.00	100.00 ± 0.00
Fulton’s condition factor (K)	1.20 ± 0.08	1.20 ± 0.06	1.17 ± 0.03

Data presented as mean ± standard deviation (SD).

## Data Availability

The 16S rRNA sequencing data generated in this study have been deposited and are openly available in the NCBI Sequence Read Archive (SRA) under the accession number PRJNA1198497 and are accessible via the following link: https://www.ncbi.nlm.nih.gov/sra/PRJNA1198497 (accessed on 25 December 2024). All other data are available upon request from corresponding authors.
